# A join point regression analysis of trends in mortality due to osteoporosis in Spain

**DOI:** 10.1038/s41598-019-40806-0

**Published:** 2019-03-12

**Authors:** Ioana Dragomirescu, Javier Llorca, Inés Gómez-Acebo, Trinidad Dierssen-Sotos

**Affiliations:** 1 School of Medicine, University of Cantabria, Santander, Spain; 2 IDIVAL, Santander, Spain; 3 CIBER Epidemiología y Salud Pública (CIBERESP), Santander, Spain

## Abstract

Osteoporosis is a major health problem in terms of fracture probability and disability. The aim of this ecological study is to identify the temporal trends in osteoporosis mortality in Spain from 1999 to 2015. Data on the Spanish population and number of deaths due to osteoporosis were obtained from the Spanish National Institute for Statistics. Age-adjusted mortality rates were estimated. Join point regression was used to identify the years when changes in mortality s and annual percentage change in mortality rates took place. Women presented a greater mortality rate decrease (p < 0.001), though this mortality difference by sex was reduced by half at the end of the period. The higher the age, the faster the mortality rate declined in women, while no clear pattern could be identified in men. In women, significant changes in trends were identified in three age groups (50–54, 60–64 and 80–84 years old). A sustained decrease in osteoporosis-associated mortality was found in women aged 75–79 and ≥85 years and men aged 60–64. In conclusion, mortality caused by osteoporosis in Spain is decreasing faster in the older age ranges especially in women.

## Introduction

Osteoporosis is defined as a systemic skeletal disease characterized by low bone mineral density (BMD) and microarchitectural deterioration of bone tissue, with a consequent increase in bone fragility and susceptibility to osteoporotic fractures which are a leading cause of morbidity and mortality worldwide^1,2^. Due to its prevalence, osteoporosis is considered a serious public health concern. It is estimated that over 200 million people suffer from this disease worldwide^3^. It affects about 27.6 million people in the European Union, the prevalence being 22.1% in women and 6.6% in men aged 50 years o over^4^. In Spain, the prevalence of osteoporosis is higher in women -reaching 26%- and lower in men -being 4.15%- and it is expected to increase with an aging population^5^. Men older than 50 present a 4.4% prevalence of hip osteoporosis and 4.8% of lumbar spine osteoporosis being much lower than in women^5^. In the USA, 2005–2008, the prevalence of osteoporosis of the lumbar spine ranges from 6.8% in women aged 50 to 59^6^. The age group ≥75 years presents a considerable difference between hip and lumbar spine osteoporosis prevalence, being 24% and 40% respectively^5^. Osteoporotic fractures are widely considered to be the most serious outcome of osteoporosis and an increased risk of death is noticeably existent in both women and men, especially after hip fracture^7^. Regarding temporal trends of osteoporotic fracture repercussions, studies in western populations have reported increases in hip fracture incidence through the second half of the 20th century; in the last two decades, however, rates seem to have stabilized while age-adjusted rates have decreased^3^. Azagra *et al*. have described that in Spain, the trend of hip fractures according to age groups and gender is clearly down, warding only women aged between 65 and 80 while this remains rather stable in the 80–84-year-old group and presents a significant increase in the 85-year-old on groups. Nevertheless, the mortality rate dropped remarkably in both sexes^8^.

Changes in the osteoporotic fracture and mortality rates trend could be related to diagnosis and treatment improvements. Regarding the diagnosis, though Dual-energy X-ray absorptiometry (DXA) is the most widely used technique to assess bone mineral, there are others that include: quantitative ultrasound, quantitative computed tomography, peripheral DXA, digital X-ray radiogrammetry, radiographic absorptiometry^9^ and magnetic resonance imaging^10^.

The WHO fracture prediction tool FRAX was created in 2008 in order to estimate fracture risk by using risk factors^11^. According to FRAX, a patient fracture risk over 10 years can be classified as low (<10% in the next 10 years), moderate (10–20% in the next 10 years), or high (>20% in the next 10 years)^12^. Other fracture risk calculators are available online^13^.

Regarding pharmacological interventions, for many years the prevention of postmenopausal bone loss was marked by the use of hormone replacement therapy (HRT)^12^ until in 2001, when Women’s Health Initiative study finding the association between HT and several cancers was published^14,15^. The widespread use in the prescription of bisphosphonates in developed countries began in 2002. Since then, an increasing use has been observed^16^. Alendronate was the first oral bisphosphonate drug for treatment of osteoporosis in 1995, followed by risedronate in 1998, and ibandronate in 2005. The generic alendronate became available in 2008 and generic ibandronate in 2012^12,17^.

The effect that the introduction of new diagnosis methods and drugs for treating osteoporosis would have had on mortality due to osteoporosis is uncertain. The main objective of this study was to evaluate trends in the mortality rate of osteoporosis in Spain from 1999 until 2015 and their relationship to changes in diagnosis and treatment in recent decades.

## Methods

### Data extraction

This ecological study used data on the number of people dying from osteoporosis divided or grouped according to sex and within a 5-year-diffeence, age groups from 50 years old on were obtained from the Spanish National Institute for Statistics, which obtained its data from national death certificates that listed osteoporosis as the cause of death. Such deaths were identified implementing, ninth revision (ICD-9) diagnosis codes, M.80 through M81.9 according to the International Classification of Diseases.

### Statistical analysis

To identify changes in mortality rate trends, join point regression was estimated for every age and sex group by using the Join point Regression Program, Version 4.5.0.1 (Statistical Research and Applications Branch, National Cancer Institute).

In brief, by using mortality rates as inputs, this method identifies the year(s) when a trend change is produced, it calculates the annual percentage change (APC) in rates between trend-change points, and it also estimates the average annual percentage change (AAPC) in the whole period studied.

To estimate the APC, the following model is used:

$\mathrm{log}({Y}_{x})={b}_{0}+{b}_{1}x$, where *log* (*Y*_*x*_) is the natural logarithm of the rate in year *x*.

Then, the APC from year *x* to year *x* + *1* is:

$$APC=\frac{{e}^{{b}_{0}+{b}_{1}(\times +1)}-{e}^{{b}_{0}+{b}_{1}\times }}{{e}^{{b}_{0}+{b}_{1}x}}\times 100=({e}^{{b}_{1}}-1)\times 100$$

When there are no join points (i.e., no changes in trend), APC is constant, so it equals the AAPC. Otherwise, the whole period is segmented by the points with trend change. Then, AAPC is estimated as a weighted average of the estimated APC in each segment by using the segment lengths as weights. For instance, in 50- to 54-year-old men, join point regression identifies two join points in 2005 and 2009, so the whole period is segmented in three periods: 1999–2005, 2005–2009, and 2009–2015, with APC equal to – 0.014, − 0.032, and – 0.012, respectively, and segment widths equal to 6, 4 and 6 years, respectively. Then, AAPC is estimated as:

$$AAPC=({e}^{\frac{-6\times 0.014-4\times 0.032-6\times 0.012}{6+4+6}}-1)\times 100=-\,1.8 \% $$

An approximate 95% confidence interval for AAPC is: (AAPC_L_,AAPC_U_), where

$$AAP{C}_{L}=({e}^{\mathrm{log}(AAPC+1)-1.96\sqrt{{w}_{x}^{2}{\sigma }_{x}^{2}}}-1)\times 100$$

$$AAP{C}_{U}=({e}^{\mathrm{log}(AAPC+1)+1.96\sqrt{{w}_{x}^{2}{\sigma }_{x}^{2}}}-1)\times 100$$

and ${\sigma }_{x}^{2}$ is the estimate of the variance of b_x_ obtained from the fit of the join point model.

The number of join points is obtained using a permutation test via Monte Carlo resampling^18^. Once the number k of join points has been obtained, the different models with k join points are compared by estimating their Bayesian Information Criterion (BIC)^19^. This procedure is detailed in the Supplementary material.

To further explore changes in trends related to events linked to diagnoses or treatment of osteoporosis in our country we have developed a point regression analysis not allowing the program to estimate the years of trend change, but pre-specifying the join points in 2003 (the year bisphosphonates were introduced) and 2008 (the year generic bisphosphonates and FRAX were introduced). Therefore, this further analysis three set periods: (1) 1999–2003 release and implementation of the Guide of Diagnosis and Treatment of Osteoporosis, (2) 2003–2008 the period of the bisphosphonates and (3) 2008–2015 the period of the generic bisphosphonates and FRAX introduction. APC for a pre-specified period is obtained as the average of the APCs previously obtained in the regular join point analysis, weighted by the number of years included in each period. This analysis has no meaning in age groups without join points: Firstly, let us further explain how it works when there is one join point: consider an age group with a join point in 2005, having APC = −1.5% in 1999–2005 and APC = −3.0% in 2005–2015. In the analysis using pre-determined join points, we will have APC = −1.5% in 1999–2003 (as −1.5 is the APC for the whole period 1999–2005), APC = [2*(−1.5%) + 10*(−3.0%)]/12, where 2 is the number of years with APC = −1.5 (i.e., 2003–2005) and 10 is the number of years with APC = −3.0% (i.e., 2005–2015). Secondly, let us apply the same procedure when there is no join point: consider an age group without join points, having APC = −2.0% in 1999–2015. In the analysis with pre-determined points the result will always be the same: APC = −2.0% in 1999–2003, APC = −2.0% in 2003–2008 and APC = −2.0% in 2008–2015. The benefit of the analysis method used allow us to identify changes affecting different age groups in different years However, a more tradittional aproach (age- and sex-adjusted mortality rates) only would allow us to identify changes affecting the population altogether. Moreover, changes in younger age groups could be undetectable -had they existed- as the impact of older age groups on age-adjusted mortality is much higher.

To further analyze mortality due to osteoporosis in Spain, an age, period and cohort analysis was performed using Poisson regression with natural cubic splines with six knots for age, five knots for period (=year of death) and three knots for birth cohort^20^.

## Results

### General trend in mortality

**Figure 1:**
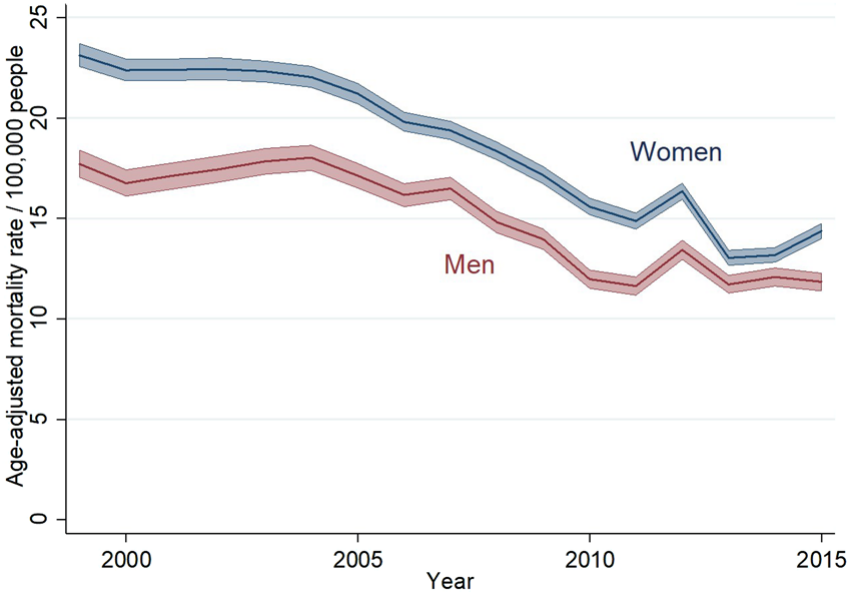
Age-adjusted mortality caused by Osteoporosis in Spain, 2000–2015; women (red line) and men (blue line).

Figure 1 displays the decreasing trend of the age-adjusted mortality rates by gender, which is more pronounced in women (p < 0.001). During the period under study, the highest osteoporosis mortality rate registered in Spain was in 1999, in both women and men (23.1/100.000 in women and 17.7/100 000 in men), decreasing around 38% in women and 33% in men at the end of the period (2015). As women presented a higher rate decrease, mortality rate differences among women and men in 2015 –at the end of the period- halved those in 1999 (5.4/10000 in 1999 vs. 2.5/100000 in 2015).

### Trend in mortality by age and sex according to the join points identified by the analysis

**Figure 2:**
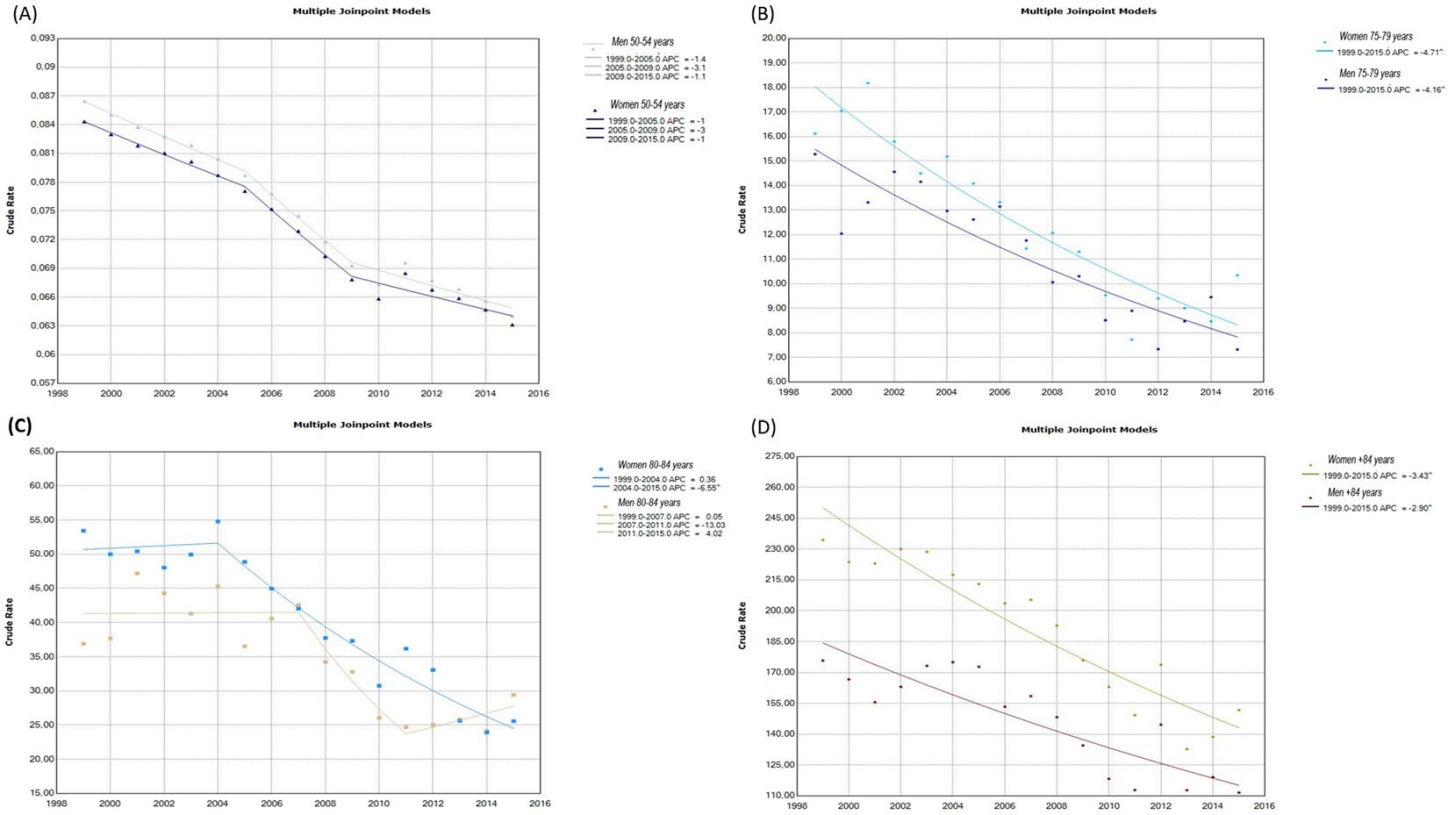
(**A**) Join point models 50–54 years. (**B**) Join point models 75–79 years. (**C**) Joinpoint models 80–84 years. (**D**) Join point models +84 years.

As osteoporosis is a disease influenced by age, in our analysis a specific analysis was made in age groups (five-year wide groups). Table 1 shows the trends in mortality caused by osteoporosis in Spain by gender, according to the join points identified by the analysis. Columns headed with AAPC indicate the trend in the whole period 1999–2015: mortality due to osteoporosis decreased in most age groups in both women and men, although such a trend was more pronounced in women over 70. Columns headed with APC display the mortality trend in each period identified by the join point regression; in five age groups (namely, 55–59, 65–69, 70–74, 75–79 and 85+), the analysis found no join point in either men or women, so APC equals AAPC. Two join points were identified in the 50–54 years old group in 2005 and 2009, leading to three periods with different trend; the fastest descendent trend in mortality was found in the intermediate period (2005/2009). Three periods (jointed in 2002 and 2009) were also identified in women, but not in men, aged 60–64; the intermediate period (2002/2009) showed a 3.4% annual decreased in mortality, while the trends were towards an increase in mortality in periods 1999/2002 and 2009/2015 (2.9% and 0.4% annual increase, respectively). Two periods were identified in the 80–84-year-old group; in women, there was an almost plain trend in 1999/2004 and a strong decrease in 2004/2015 (−6.5% annual change), while in men the decrease began in 2007 (−13% decrease in 2007/2011), but it was followed by an increase in 2011/2015. Results from selected age groups are displayed in Fig. 2.

**Table 1:** Trends in mortality caused by Osteoporosis in Spanish women and men: Year of change of trend, annual percentage change, and annual average percentage change.

Age group, yrs	Period	Women			Period	Men		
		Change year	APC (95% CI)	AAPC (95% CI)		Change year	APC (95%CI)	AAPC (95% CI)
50–54	1999–2015			−1.7 (−2.3,−1.1)	1999–2015			−1.8 (−2.3, −1.3)
	1999–2005	2005	− 1.4 (−2.1,−0.7)		1999–2005	2005	−1.4 (−2.1, −0.8)	
	2005–2009	2009	− 3.2 (−5.2,−1.1)		2005–2009	2009	− 3.2 (−5.0, −1.3)	
	2009–2015		− 1.0 (−1.8,−0.3)		2009–2015		− 1.2 (−1.8, −0.5)	
55–59	1999–2015	None	+1.2 (−3.5, +6.2)	+1.2 (−3.5, +6.2)	1999–2015	None	− 1.5 (−3.8, +1.0)	−1.5 (−3.8, +1.0)
60–64	1999–2015			−0.8 (−1.3, −0.3)	1999–2015	None	−5.1 (−8.9, −1.2)	−5.1 (−8.9, −1.2)
	1999–2002	2002	+2.9 (+0.6, +5.1)					
	2002–2009	2009	−3.4 (−4.1, −2.7)					
	2009–2015		+0.4 (−0.3, +1.2)					
65–69	1999–2015	None	−2.5 (−7.7, +3.1)	−2.5 (−7.7, +3.1)	1999–2015	None	+0.5 (−5.5, +6.9)	+0.5 (−5.5, +6.9)
70–74	1999–2015	None	−4.8 (−10.8, +1.6)	−4.8 (−10.8, +1.6)	1999–2015	None	−0.4 (−4.3, +3.6)	−0.4 (−4.3, +3.6)
75–79	1999–2015	None	−4.7 (−5.7, −3.7)	−4.7 (−5.7, −3.7)	1999–2015	None	−4.2 (−5.2, −3.1)	−4.2 (−5.2, −3.1)
0–84	1999–2015			−4.4 (−5.9, −3.0)	1999–2015			−2.4 (−6.5, +1,8)
	1999–2004	2004	+0.4 (−4.0, +5.0)		1999–2007	2007	+0.1 (−3.4, 3.6)	
	2004–2015		−6.5 (−7.9, −5.2)		2007–2011	2011	−13.0 (−25.7, +1.8)	
					2011–2015		+4.0 (−5.7, +14.7)	
85+	1999–2015	None		−3.4 (−4.2, −2.7)	1999–2015	None		−2.90 (−3.8, −2.0)

APC = annual percent change; CI = confidence interval. AAPC = annual average percent change; CI = confidence interval.

### Trend in mortality defined by age and sex in pre-specified periods

To explore the influence of changes in treatment or diagnosis on mortality trends, we have estimated the average APC in three predefined periods (Table 2): the first one (1999–2003, before bisphosphonate introduction) presented only a 1.4% decrease both in women and men in the 50–54 years age group. Nevertheless, in the second period (2003–2008, after bisphosphonates introduction) the same age group presented a more pronounced decline in both genders compared with the previous period (−2.5 95% CI −3.6 to −1.3 in women and −2.5% 95% CI −3.5 to −1.5 in men). In addition, women in the 80–84 years age group showed the higher significant decline (−5.2% (95% CI −6.4 to 4.0)). Finally, between 2008–2015 (third period, after generic bisphosphonates and FRAX introduction) in the 50–54 age group the decrease was 1.3% in women and 1.5% in men, the 6.5% decrease observed in women in the 80–84 years age group is worth highlighting.

**Table 2:** Trends in mortality with pre-designed join point caused by Osteoporosis in Spanish men and women.

Age group, yrs	Period	Women	Period	Men
		AAPC (95% CI)		AAPC (95% CI)
50–54	1999–2003	−1.4 (−2.1, −0.7)	1999–2003	−1.4 (−2.1, −0.8)
	2003–2008	−2.5 (−3.6, −1.3)	2003–2008	−2.5 (−3.5, −1.5)
	2008–2015	−1.3 (−1.9, −0.7)	2008–2015	−1.5 (−2.0, −0.9)
60–64	1999–2003	+1.2 (− 0.2, +2,7)		
	2003–2008	− 3.4 (− 4.1, −2.7)		
	2008–2015	− 0.1 (− 0.7, +0.4)		
80–84	1999–2003	+0.4 (−4.0, +5.0)	1999–2003	+0.1 (−3.4, +3.6)
	2003–2008	−5.2 (−6.4, −4.0)	2003–2008	−2.7 (−6.2, +0.9)
	2008–2015	−6.5 (−7.9, −5.2)	2008–2015	−3.7 (−10.7, +3.9)

AAPC = annual average percent change; CI = 95% confidence interval.

*p < 0.05.

Of note, this analysis could only be carried out on those age groups which are in Table 1 and identified at least one join point (i.e.: at least two periods in mortality trend).

The age-period-cohort analysis did not find any period or cohort effect in neither women nor men. Its results are displayed in Supplementary Table 1 and Supplementary Fig. 1.

## Discussion

According to our results, mortality rates by osteoporosis are decreasing in both Spanish women and men, the decline being faster in women aged over 70. Although we identified different trend periods in some age groups (namely, 50–54, 60–64, 80–84 years old), it is unclear whether the introduction of new diagnosis/screening techniques such-as FRAX or DXA- or new drugs such-as bisphosphonates- could have played a role in the mortality trends we have described.

For a specific measure to be considered responsible for changes in mortality trend, one could have expected some age-related pattern emerging in the analysis. For instance, if alendronate had been generalized in women aged 70 onwards from, say, 2003, then a faster mortality decline could have been expected in women included in all age groups over 70. Our analysis, however, does not support the existence of any clear pattern, as only three non-consecutive age groups experienced trend changes, while most age groups had a sustained decrease in mortality throughout the whole studied period. The fact that 11 out of 16 groups, organized or defined by age and sex have no join points strongly suggests that there has been no change in the study period.

Several factors would explain this lack of accelerated declining pattern after implementing new diagnosis/treatments. Firstly, the low adherence to both osteoporotic treatment at the time of fracture occurrence and DXA testing^21^; secondly, a great variability observed in osteoporosis treatment among general practitioners, who prescribe medication to a high percentage of women without a high FRAX risk while keeping those women with a high FRAX risk untreated^22^. Lastly, in our analysis, we have not considered a lag period; i.e.: there could have been a delay between the introduction of new measures for reducing or treating osteoporosis and any effect of them on mortality.

Some results of ours are, however, noteworthy. The steepest decline observed was around 5% in specific age groups (75–79 years old women and 60–64 years old in men). Along the same lines, Azagra *et al*., studying hip fractures (the most serious cause of mortality) found that in women aged between 75–79 years, the incidence rate fell significantly by 7.7%^8^. In the same way, men in the 75–79 years old group presented a globally significant decrease that could be related to the recommendation of DXA in men from 70 years on^23^. Furthermore, screening rates were higher among men older than 75 years^24^.

In age groups where trend changes were identified, the largest decline in incidence rate was observed in women aged 80–84 years old in the 2004–2015 period. This trend is consistent with the decrease observed in osteoporosis diagnosis (from 73% to 69%) between 2002 and 2012^25^. In this period, oral bisphosphonate initiation shifted towards older women and those with prior fractures^26^, which corresponds to the increasing focus on primary and secondary fracture prevention of patients at elevated fracture risk given by WHO’s FRAX introduction. In addition, this age group is likely to represent patients with polypharmacy which is associated with better treatment adherence^27^. On the other hand, the lack of significant changes in tendency observed in men could be related to the fact that men were less likely to receive osteoporosis treatment (8%) compared with women (23.3%) after a hip fracture, as observed in a study between 2000 and 2010^28^. Our results are consistent with a meta-analysis published by Bolland *et al*., which shows a 10% decrease of the mortality risk associated with osteoporosis treatment in the older population^29^. However, an ecological studies developed in our country has failed to show correlation between the increasing use of antiresorptive therapy and the incidence of femoral fracture^30^.

On the other hand, in the youngest group (50–54 years) the greatest decrease in mortality was observed in the 2005–2009 period, which could be related to the decline of the prescription of the HRT^14,15^ and the increasing use of bisphosphonates^16^. On the contrary, in the period 2009–2015 a slow decrease in th mortality rate was observed. We can specula that these changes in trend might be associated with the release of generic bisphosphonates^14,31^. Regarding this point, in February 2008 the brand alendronate patent expired and generic alendronate became available; four years later (2012) ibandronate was marketed as generic^17^. At the same time, practice guidelines in the UK and elsewhere recommended that generic alendronate should be viewed as the first-line treatment and this currently dominates many European markets^32^. Since the introduction of generic bisphosphonates, reports have consistently concluded that its adherence is poorer than the original brand^32–34^, which could eventually lead to higher rates of gastro-intestinal intolerance^35^, lower increase of lumbar spine and total hip bone mineral density^36,37^. In addition, age group 50–64 years present high level of treatment and low prevalence of risk factors^38,39^. In Spain, primary treatment has been associated with lower adherences than secondary treatment^27^. Treatment adherence represents a common problem in the treatment of osteoporosis^34^ and it is responsible for an increased risk of fracture of approximately 30%^32^ and increases the cost-effectiveness ratio of osteoporosis screening strategies^26^.

Finally, the different trends observed in groups organized by sex in the 60–64 year olds group must be stressed. In men, a sharp decline of mortality trends was detected through the whole period, while women showed a less pronounced decrease. This finding does not seem to be related to changes in bisphosphonates treatment, given that men were less likely to receive osteoporosis treatment after a hip fracture compared with women^28^. However, the improvement of the evaluation and treatment of glucocorticoid-induced osteoporosis (the most common cause of secondary osteoporosis in men) could be related to this descendent trend^40,41^. On the other hand, two significant join points were detected in women between 60–64 years old, showing opposite trends: an increase of mortality rate at the early years (1999–2002) and a markedly subsequent decline (2002–2009). The initial increase was probably related to the fact that hormone therapy was the most commonly prescribed treatment in postmenopausal women before 2001^12^, while the later decline could probably be due to the introduction of bisphosphonates from 2002 despite the decline of the proportion of women under 65 years old meeting treatment criteria applying FRAX since 2008^11^.

In addition to the new diagnostic methods and treatments, some articles in the United States^42^, Canadá^43^, Sweden^44^, Denmark^45^, Portugal^46^ and Korea, suggest that trends in mortality from osteoporosis could be motivated by a birth cohort effect. For example, the economic or political situation of a country that leads to better or worse maternal nutrition and the nutrition of children^47^. However, few articles have evaluated cohort and period effects and their results are contradictory regarding the period effect^43,44,48–50^. However, recent studies suggest that the birth cohort effect and the period effect suppose a significant reduction in the incidence of hip fracture in each cohort or subsequent birth period and these effects are more marked among women than among men^43,44^. In our article, we have found no birth cohort effect on death due to osteoporosis.

Some reproductive factors observed in United States such as the increase in the average number of reproductive years^51^, are not sustained either, given that they do not explain the parallel changes in the decrease in hip fractures in men. Similarly, other authors consider other factors such as an increase in calcium and vitamin D intake^52^, a higher BMI^53^, greater physical activity, smoking cessation^51^ and the prevention of falls^54^ to explain the decreasing trends in the incidence of hip fracture. It may contribute to the decline of hip fractures and therefore the decline of mortality due to osteoporosis. New investigations are necessary to prove it. Despite the high prevalence of osteoporosis in older population, its impact on mortality has scarcely been studied. To the best of our knowledge, this is the first study evaluating trends on mortality caused by osteoporosis developed in Spain. However, our study also has some limitations. Firstly, Join point regression consists in an ecological study, so causal relationship cannot be established and our results require further confirmation with individual-level data. As such, we can only hypothesize about associations highlighted by our data and have strayed from making claims of causality. Secondly, databases of the Spanish National Institute of Statistics do not provide osteoporosis classification data so we are not able to determine which data corresponds to primary osteoporosis or secondary one. Thirdly, the scarce number of studies focused on osteoporosis mortality makes it difficult to compare our results and forces us to contrast them with studies focused on osteoporotic fractures. However, we consider osteoporotic fractures an acceptable proxy to mortality because it has been demonstrated that general fractures, and especially hip fractures, are related to reducing personal autonomy through disability and dependence^55^, influencing the quality of life^56,57^ and even mortality^58,59^.

In conclusion, osteoporosis mortality in Spain is decreasing faster in the older age cohorts especially in women; the causes of this are not clear, but it cannot be fully attributed to improvements in osteoporosis diagnosis or screening or in primary or secondary osteoporosis prevention via bisphosphonates. Further observational studies with individual-level data are needed to clarify if new diagnosis, preventative tools, or their combination could contribute to these changes on mortality due to osteoporosis.

## Supplementary information

Supplementary material
