# Supplementary material for: A join point regression analysis of trends in mortality due to osteoporosis in Spain
Source: Sci Rep. 2019 Mar 12;9:4264. doi: 10.1038/s41598-019-40806-0 (PMC6414692; doi:10.1038/s41598-019-40806-0)

**A join point regression analysis of trends in mortality due to osteoporosis in Spain**

**Short title: Trends in mortality due to osteoporosis in Spain**

Authors: Ioana Dragomirescu^1^, Javier Llorca^1,2,3^, Inés Gómez-Acebo^*,1,2,3^, Trinidad Dierssen-Sotos^*,1,2,3^

*Inés Gómez-Acebo and Trinidad Dierssen-Sotos share senior authorship.

Affiliations:

1: School of Medicine, University of Cantabria, Santander, Spain.

2: IDIVAL, Santander, Spain.

3: CIBER Epidemiología y Salud Pública (CIBERESP), Spain.

**Supplementary material:**

**Detailed methods for identifying the number of join points and the final model**

In order to identify the number of join points, a permutation test via Monte Carlo resampling is implemented by comparing the sum of squared errors (SSE) of a null model with k_N_ join points and the SSE of an alternative model with k_A_ join points, where k_A_>k_N_. It begins with k_N_ = 0 and k_A_ = the pre-specified max. If the null hypothesis is rejected, then k_N_ is set at k_N+1_; if the alternative hypothesis is rejected, then k_A_ is set to k_A-1_. The procedure continues until k_N_ = k_A_ = k, is the final number of join points^18^.

Once the number k of join points has been obtained, the different models with k join points are compared by estimating their Bayesian Information Criterion (BIC) as:

$BIC\left( k \right)=ln\frac{SSE(k)}{n}+2(k+1)\frac{ln(n)}{n}$ , where n is the number of observations and 2(k+1) is the number of parameters in the model. The model with k join points with the minimum modified-BIC^19^ is selected, where the modified-BIC is:

$MBIC\left( k \right)=BIC\left( k \right)+\frac{ln\left| {X^{'}}_{k}(\hat{\tau})X_{k}(\hat{\tau)} \right|}{n}-\frac{2}{n}ln\text{}\left( \frac{n-k-3}{2} \right)-\frac{k+3}{n}ln(SSE\left( k \right))$,

where Γ(z) is the gamma function: $\text{}\text{(z)=}\int_{0}^{\infty} t^{z-1}e^{-t}dt$ and

$$X_{k}\left( \hat{\tau} \right)=\left( \begin{aligned} 1 x_{1} \left( x_{1}-\hat{\tau_{1}} \right)^{+} \cdots\left( x_{1}-\hat{\tau_{k}} \right)^{+} \\ \vdots\vdots\vdots\ddots\vdots\\ 1 x_{n} \left( x_{n}-\hat{\tau_{1}} \right)^{+} \cdots\left( x_{n}-\hat{\tau_{k}} \right)^{+} \end{aligned} \right)$$

x_i_ are the values of the independent variable, $\hat{\tau_{j}}$ are the estimated joinpoints and a^+^ = max(a,0).

Supplementary Table 1. Age-period-cohort analysis on mortality due to osteoporosis in Spain

|  | **Women** | | | | **Men** | | | |
| --- | --- | --- | --- | --- | --- | --- | --- | --- |
|  | **Coef** | **95% Conf. Interval** | | **p** | **Coef** | **95% Conf. Interval** | | **p** |
| **Age** | 0.0398 | 0.0243 | 0.0552 | <0.001 | 0.0388 | 0.0283 | 0.0494 | <0.001 |
| **A1** | -0.0002 | -0.0009 | 0.0005 | 0.536 | -0.0002 | -0.0008 | 0.0005 | 0.645 |
| **A2** | 0.0007 | -0.0002 | 0.0016 | 0.135 | 0.0006 | -0.0003 | 0.0015 | 0.218 |
| **A3** | -0.0015 | -0.0025 | -0.0006 | 0.001 | -0.0012 | -0.0021 | -0.0002 | 0.017 |
| **A4** | 0.0027 | 0.0018 | 0.0036 | <0.001 | 0.0020 | 0.0011 | 0.0030 | <0.001 |
| **A5** | -0.0030 | -0.0037 | -0.0022 | <0.001 | -0.0022 | -0.0029 | -0.0015 | <0.001 |
| **A6** | 0.0015 | 0.0011 | 0.0020 | <0.001 | 0.0010 | 0.0006 | 0.0014 | <0.001 |
| **P1** | -0.0208 | -0.0557 | 0.0142 | 0.244 | -0.0035 | -0.0420 | 0.0351 | 0.860 |
| **P2** | 0.0115 | -0.0296 | 0.0527 | 0.582 | -0.0203 | -0.0669 | 0.0263 | 0.393 |
| **P3** | -0.0018 | -0.0343 | 0.0306 | 0.911 | 0.0343 | -0.0037 | 0.0723 | 0.077 |
| **P4** | 0.0097 | -0.0139 | 0.0334 | 0.420 | -0.0154 | -0.0439 | 0.0131 | 0.290 |
| **P5** | -0.0123 | -0.0270 | 0.0025 | 0.104 | -0.0035 | -0.0214 | 0.0145 | 0.705 |
| **C1** | -0.0005 | -0.0010 | 0.0000 | 0.067 | -0.0002 | -0.0005 | 0.0002 | 0.336 |
| **C2** | 0.0006 | -0.0001 | 0.0012 | 0.079 | 0.0003 | -0.0002 | 0.0007 | 0.248 |
| **C3** | -0.0003 | -0.0006 | 0.0000 | 0.096 | -0.0002 | -0.0004 | 0.0001 | 0.184 |

A1-A6: knots for age effect. P1-P5: knots for period effect. C1-C3: knots for cohort effect

Supplementary figure 1. Age-period-cohort analysis on mortality due to osteoporosis in Spain. (a), (b) and (c): Women. (d), (e) and (f): Men. (a) and (d) are age-specific mortality rates if period and cohort relative risks are 1. (b) and (e) are relative risks for period. (c) and (d) are relative risks for birth cohort.


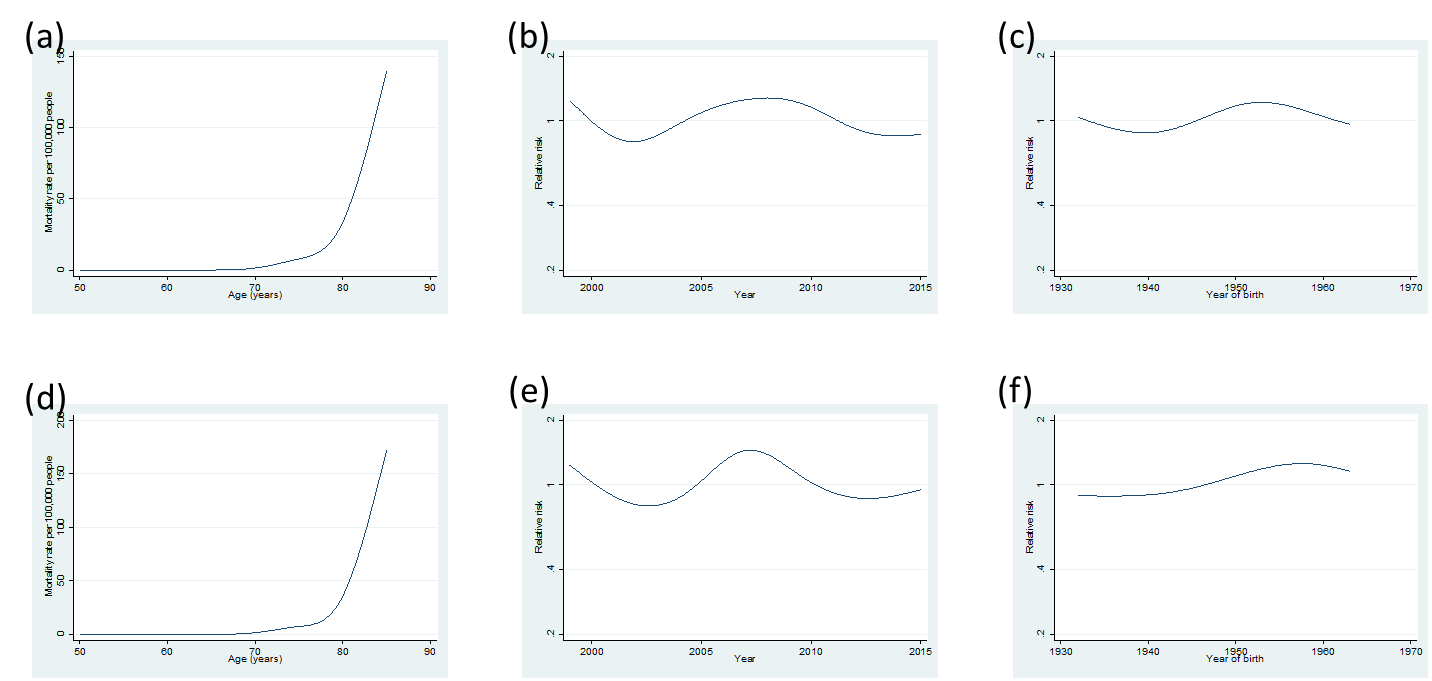

Supplement: Supplementary file 1 — Supplementary material [file 41598_2019_40806_MOESM1_ESM.docx]
